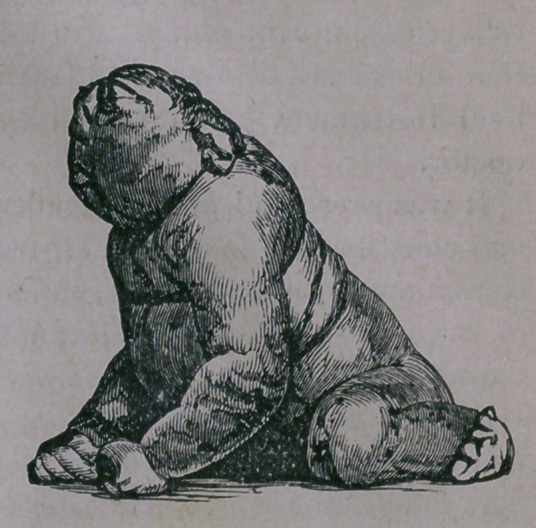# Remarkable Case of Monstrosity, with Cut

**Published:** 1869-12

**Authors:** E. R. Hutchins

**Affiliations:** Philadelphia


### Original Communications.

ARTICLE I.—*A Case of Monstrosity.* By E. R. HUTCHINS, M.D., Philadelphia.

I SEND you, herewith, a photograph of one of those curious cases of monstrosity, classed under the head of akephalous. The history of the case is, briefly, as follows:

Mrs. G., aged thirty-seven, and mother of three children, has had four miscarriages. She had been under my charge for three months prior to her becoming pregnant, for the treatment of profuse menorrhagia, dependent upon ulceration of os and cervix uteri. Eight months subsequently I was summoned to her bedside, and found her excessively nervous, and complaining of dull, spasmodic pain, simulating labor pains, and of a constant discharge of water from the vagina. Upon examination *per vaginam*, I found the os soft and flabby, but not open larger than a ten-cent piece (if you remember, dear editor, how large these are), but with a dribbling of colored water, without any offensive odor. I ordered her ten grains of bromide of ammonium every four hours, and left her.

Next day, no change. Forty-eight hours after, her pains became more decided, and with shorter intervals, and upon examination the os was found widely open, and a soft, spongy mass pushing its way down through the strait. The labor was protracted; but after eleven hours, the original of the picture accompanying this was ushered into the world—dead. It could not have been dead long, as it presented no sign of putrefaction.

The *basis-cranii* was badly shaped, and the membrane covering it was continuous with the integuments. There was an entire absence of bones at the side and upper part of the cranium, and a total want of brain or membranes. As can be seen from the picture, the skull runs directly backwards from the superciliary ridge. The specimen was absolutely larger than the natural, full-formed fœtus. There was absence, also, of the spinal marrow. The Berlin theory of Prof. Rudolphi, that which accounts for the akephaloid monster, by its origin in *hydrokephalus*, would seem to be borne out in this case, as it has in many others, viz.: by the great abundance of liquor amnii. In this case there was an uninterrupted flow for over fifty hours. The mother had a speedy and excellent recovery.

The all-absorbing theme in our profession, and indeed among the *laity* recently, has been the question of *female attendance* at *our clinics*. As the matter is still under discussion, I reserve an account of it till my next communication.

## Figures and Tables

**Figure f1:**